# Genome-wide Regulatory Roles of the C2H2-type Zinc Finger Protein ZNF764 on the Glucocorticoid Receptor

**DOI:** 10.1038/srep41598

**Published:** 2017-01-31

**Authors:** Abeer Fadda, Najeeb Syed, Rafah Mackeh, Anna Papadopoulou, Shigeru Suzuki, Puthen V. Jithesh, Tomoshige Kino

**Affiliations:** 1 Division of Translational Medicine, Sidra Medical and Research Center, Doha 26999, Qatar; 2 Division of Biomedical Informatics, Sidra Medical and Research Center, Doha 26999, Qatar; 3 Program in Reproductive and Adult Endocrinology, Eunice Kennedy Shriver National Institute of Child Health and Human Development, National Institutes of Health, Bethesda, MD 20892, USA; 4 Department of Pediatrics, Asahikawa Medical University, Asahikawa 078-8510, Japan

## Abstract

The C2H2-type zinc finger protein ZNF764 acts as an enhancer for several steroid hormone receptors, and haploinsufficiency of this gene may be responsible for tissue resistance to multiple steroid hormones including glucocorticoids observed in a patient with 16p11.2 microdeletion. We examined genome-wide regulatory actions of ZNF764 on the glucocorticoid receptor (GR) in HeLa cells as a model system. ZNF764- and GR-binding sites demonstrated similar distribution in various genomic features. They positioned predominantly around 50–500 kbs from the transcription start sites of their nearby genes, and were closely localized with each other, overlapping in ~37% of them. ZNF764 demonstrated differential on/off effects on GR-binding and subsequent mRNA expression: some genes were highly dependent on the presence/absence of ZNF764, but others were not. Pathway analysis revealed that these 3 gene groups were involved in distinct cellular activities. ZNF764 physically interacted with GR at ligand-binding domain through its KRAB domain, and both its physical interaction to GR and zinc finger domain appear to be required for ZNF764 to regulate GR transcriptional activity. Thus, ZNF764 is a cofactor directing GR transcriptional activity toward specific biologic pathways by changing GR binding and transcriptional activity on the glucocorticoid-responsive genes.

Steroid hormones exert diverse physiologic functions and play central roles in human physiology^1^,^2^. Among them, glucocorticoids are critical for the maintenance of organ homeostasis and adaptive response against circumstance changes^3^,^4^. On the other hand, sex steroids including androgens are necessary for gender-specific development of reproductive organs in fetal and adolescent phases and acquisition/maintenance of fertility and other functions during reproductive ages^5^,^6^,^7^,^8^. These diverse actions of steroid hormones are mediated by the specific intracellular receptors of the steroid hormone receptor (SR) family, such as the glucocorticoid (GR) and the androgen (AR) receptor^9^. SRs consist of common structural domains, the N-terminal immunogenic (NTD), middle DNA-binding (DBD) and C-terminal ligand-binding (LBD) domain^9^. Their DBD consists of two C4-type zinc fingers with which they physically interact as a dimer with an inverted hexameric palindrome separated by 3 base pairs^9^,^10^. LBD of these receptors is composed of 12 α-helices and 4 β-sheets, and has a ligand-binding pocket created with its helices^9^,^11^. These receptors share some DNA-binding motifs, as they maintain high sequence homology in their DBDs due to phylogenic proximity^9^. Ligand-activated and DNA-bound SRs modulate their transcriptional activity by attracting and/or communicating with cofactors, other transcription factors and chromatin-associated molecules through their two transactivation domains, activation function (AF)-1 and -2^9^,^12^. AF-1 is located in NTD and its activity is ligand-independent, while AF-2 is created on LBD through the ligand-induced conformational change of this domain where intramolecular shift of the helix-12 plays a critical role^13^. Core AF-2 directly binds the LxxLL motif located in the nuclear receptor box (NRB) of the p160-type histone acetyltransferase coactivators or nuclear receptor coactivators (NCoAs) for attraction of these cofactors to the promoter region of glucocorticoid-responsive genes^9^,^14^. Importantly, SRs share some of these cofactor molecules for transcriptional regulation^15^, thus defects in one such protein may potentially influence the transcriptional activity of several SRs, and could develop pathologies that span over multiple hormones.

In agreement with this hypothesis, one family with resistance to glucocorticoids, mineralocorticoids and androgens was previously reported, with speculation of a congenital coactivator defect as a primary cause^16^,^17^. We also reported one case with 16p11.2 microdeletion who demonstrated partial resistance to glucocorticoids, androgen, and possibly, thyroid hormones^18^. We found that haploinsufficiency in the zinc finger protein (ZNF) 764 gene located in the deleted area appears to be responsible for his multiple steroid hormone resistance^18^. ZNF764 is a member of the Krüppel-type zinc finger protein (ZNF) family (KRAB-ZNF), which consists of over 800 proteins that act as DNA-binding transcriptional regulators^19^. Typical members of this family consists of one Krüppel-associated box (KRAB) domain and multiple C2H2-type zinc fingers in its N- and C-terminal portions, respectively^19^,^20^. The KRAB domain, which can be divided into A and B boxes, interacts with the KRAB-associated protein 1 (KAP1), a large cofactor protein also known as the transcriptional intermediary protein 1β (TIF1β), the KRAB-A-interacting protein 1 (KRIP-1) or the tripartite motif-containing 28 (TRIM28), and mediates transcriptional regulatory actions (both activation and repression) of the KRAB-ZNF proteins^20^,^21^,^22^. On the other hand, multiple zinc fingers bind target DNA sites in a sequence-specific fashion^23^. One C2H2-type zinc finger consists of one α-helix and two β-sheets and the former lies into the major groove of the DNA double helix, resulting in wrapping around the DNA polymer with multiple fingers^24^,^25^. In addition, some of the KRAB-ZNFs use their zinc fingers for interacting with other proteins or double-stranded RNAs^26^,^27^,^28^,^29^. KRAB-ZNFs are vertebrate specific, are highly variable in their numbers with frequent gene duplication/deletion, and their family members have expanded following evolution, indicating their huge diversification, and thus, a prominent role in adaptive evolution, particularly in higher organisms including humans^30^,^31^. Thus, KRAB-ZNFs appear to be critical transcription factors for the development/organization of species-specific characteristics/functions in humans by regulating spatio-temporal expression of certain groups of genes in respective organs^23^,^30^.

In our previous study, we found that ZNF764, which is a 407 amino acid protein with one KRAB-A domain and seven C2H2-type zinc fingers respectively in its N- and C-terminal half, functions as a potent coactivator of several SRs^18^. We therefore examined genome-wide regulatory roles of ZNF764 on SR-induced transcriptional activity by employing GR as a model system. We found that ZNF764 and GR interact with DNA closely, and ZNF764 differentially modulates DNA-binding profiles of GR and directs its transcriptional activity toward specific biologic pathways through physical interaction.

## Results

To examine genome-wide regulatory actions of ZNF764 on GR, we performed chromatin immunoprecipitation (ChIP) reactions followed by high-throughput sequencing (ChIP-Seq) using anti-GR or -ZNF764 antibody in HeLa cells transfected with control or ZNF764 small interfering RNA (siRNA) and treated with or without the synthetic glucocorticoid dexamethasone. We also performed RNA-Seq to find glucocorticoid-responsive genes in these cells. We identified statistically significant ZNF764- and GR-binding sites (or peaks). As a measure of quality, the fraction of reads (FRiP) in the peaks of these molecules was > 1% (Supplemental Table 1), which fulfilled the quality standard recommendation by the Encyclopedia of DNA Elements consortium (ENCODE), thus our data were suitable for further analyses. We found that ZNF764 and GR respectively had statistically significant (p < 0.01) 3,639 and 1,906 peaks in the presence of dexamethasone.

### ZNF764 and GR bind DNA in close proximity and distantly from the transcription start site (TSS)

**Figure 1:**
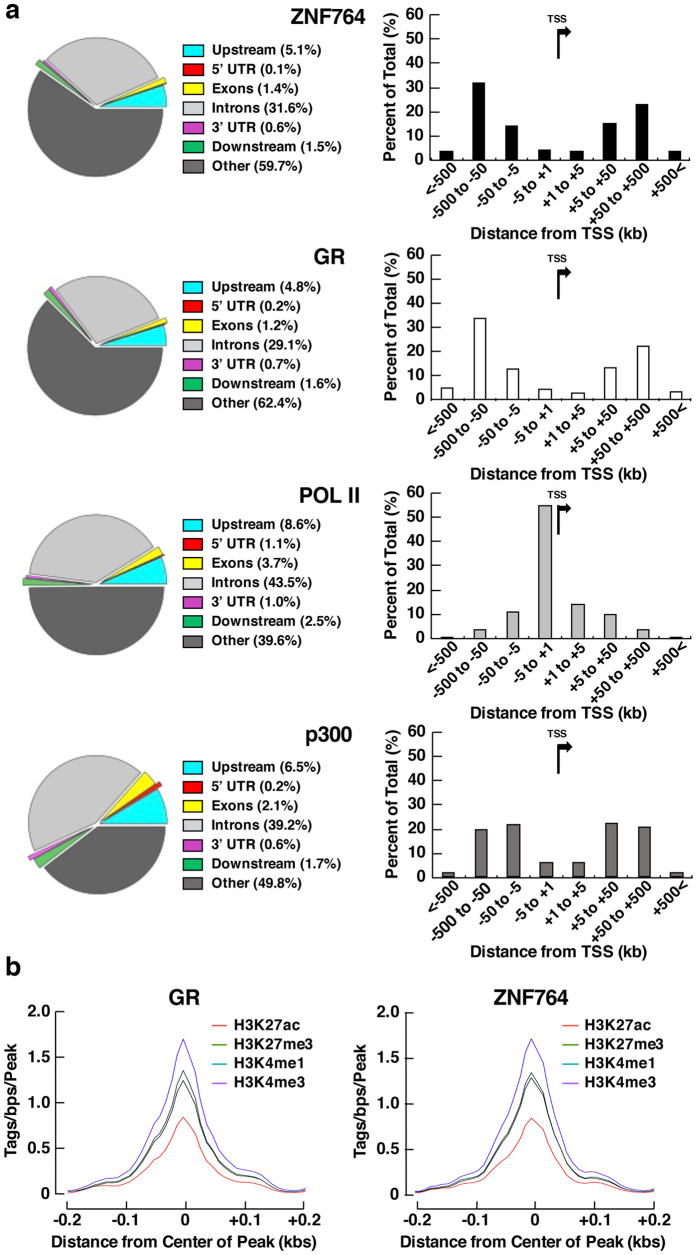
ZNF764 and GR demonstrate similar genomic DNA-binding profiles in HeLa cells. (**a**) ZNF764- and GR-binding sites are associated with similar genomic features, which are distinct from those for the RNA polymerase II- and p300-binding sites. Distribution of the binding sites for ZNF764, GR, RNA Polymerase II (POLII) and p300 in known genomic features are shown in the left panels, whereas their distribution with regard to the distance from TSSs of the closest genes are shown in the right panels. TSS: Transcription start site, POLII: RNA polymerase II, UTR: untranslated region. (**b**) ZNF764- and GR-binding sites are associated with similar histone marks. Distribution of histone marks (H3K4me3: purple, H3K4me1: blue, H3K27ac: orange and H3K27me3: green) associated with ZNF764- or GR-binding sites are shown.

We first mapped ZNF764- and GR-binding sites in major genomic features, such as exons, introns, 5′ or 3′ untranslated regions (UTRs), and upstream or downstream portions of the protein-coding genes (Fig. 1a, left panels). We compared them to the binding sites of the RNA polymerase II (POLII) and the general transcriptional coactivator p300 by obtaining their corresponding data for HeLa cells from ENCODE. ZNF764 and GR demonstrated similar distribution profiles of their binding sites with their majority falling outside the gene-coding regions, such as exons, introns and UTRs, in contrast to POLII and p300. We then examined the distribution of ZNF764- and GR-binding sites with respect to the distance from TSSs of the closest genes, and found that they were located mostly in the region >5 kbs away from TTSs, again demonstrating similar distribution profiles (Fig. 1a, right panels). Specifically, ~60% of their binding sites were positioned within the 50–500 kbs area (more in distal), located either upstream or downstream of TSSs. In contrast, the majority of the POLII-binding peaks lied within 5 kbs from TSSs (mostly upstream), whereas those of p300 were identified within the 5–500 kbs area. These results indicate that ZNF764 and GR bind at similar distant genomic regions of the nearby genes, the pattern of which is distinct from that of the POLII or p300. We further examined association of ZNF764- and GR-binding sites to the genome regions harboring specific chromatin modification patterns correlating with distinct functionality, such as the histone (H) 3, lysine (K) 4 trimethylation (H3K4me3): active promoters, H3K4me1: enhancers, H3K27 acetylation (ac): enhancer/promoter activation and H3K27me3: polycomb repression. We found that their binding sites were coupled with comparable histone marks (Fig. 1b), suggesting that ZNF764 and GR bind to the similar genome regions with characteristic activities.

### ZNF764 binds DNA very closely to GR

**Figure 2:**
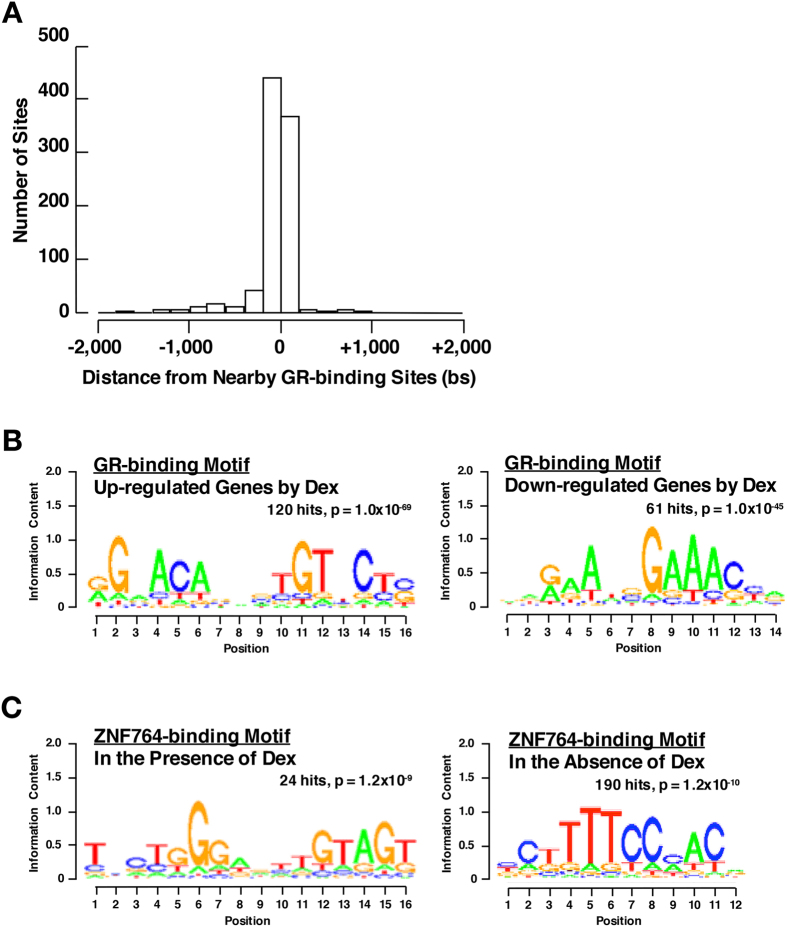
ZNF764- and GR-binding sites are located in close proximity. (**A**) Distribution of ZNF764-binding sites to the nearby GR-binding peaks in HeLa cells. (**B**) GR-binding sites found with the genes up-regulated and down-regulated by dexamethasone are associated respectively with a classic tandem GREs motif and a *de novo* motif. The DNA sequences within 600 bps from the peak center of all GR-binding sites found with dexamethasone up-regulated or down-regulated genes were used for the motif analysis. (**C**) ZNF764-binding sites found in the presence and absence of dexamethasone are respectively associated with the motifs previously reported for BCL6 and ZNF75-A. The analysis was respectively limited to the 100 and 190 ZNF764-binding sites with highest confidence (lowest p values).

The above results prompted us to examine the distance between ZNF764- and GR-binding sites. We found that most of the identified ZNF764-binding sites were located within the region around −200 to +200 kbs from the nearby GR-binding sites (Fig. 2A). Indeed, 1,340 ZNF764-binding sites [36.9% of all sites (3,639)] overlapped with those of GR, suggesting that ZNF764 either binds DNA very closely to GR or is among the same protein complexes formed with GR on DNA.

We explored DNA-binding motif(s) of the GR or ZNF764 in their respective binding sites. For GR, we were able to retrieve the classic tandem GREs motif for the genes up-regulated by dexamethasone, consistent with several previous reports^32^,^33^,^34^,^35^ (Fig. 2B, left panel). In contrast, we identified a *de novo* GR-binding motif for the genes down-regulated by this steroid, which is distinct from the reported motifs as well as from the novel negative GREs characterized by two inverted repeats with 0-2 spacers^34^,^36^ (Fig. 2B, right panel). Finding a *de novo* ZNF764-binding motifs was illusive; We found 9-10 dinucleotide repeat (AC/G) in the presence of dexamethasone (found in 2,703 ZNF-binding sites). Since C2H2-type zinc fingers usually recognize tri-nucleotide repeats, the dinucleotide repeats we found do not appear to be true binding motifs^23^. Therefore, we limited the analysis to the top 100 ZNF764-binding sites with highest confidence (lowest p values), and found a motif previously reported for the B-cell CCL/lymphoma (BCL6) in the presence of dexamethasone (Fig. 2C, left panel). BCL6 is the C2H2-type ZNF protein harboring one BTB/POZ domain and nine zinc fingers respectively in its N- and C-terminus^37^. In the absence of dexamethasone, we identified the ZNF75-A-binding motif in the top 190 ZNF-binding sites with highest confidence (Fig. 2C, right panel). ZNF75-A is a KRAB-ZNF protein similar to ZNF764, and contains five C2H2-type zinc fingers^38^.

### Presence or absence of ZNF764 differentially regulates GR’s binding to DNA and transcriptional activity on the glucocorticoid-responsive genes

We examined the effect of ZNF764 on GR’s binding to DNA by knocking down the former with its siRNA in HeLa cells. ZNF764 siRNA significantly reduced the ZNF764 protein levels in these cells (Supplemental Figure 1). We found 1,175 GR-binding sites in 529 genes under ZNF764 KD compared to 1,906 binding sites in 627 genes with control KD (Table 1). Ratios of the dexamethasone up-regulating genes vs. down-regulating genes were 0.97 (67 out of 69 genes) under ZNF764 KD and 0.88 (37 out of 42 genes) under control KD, indicating that most of the glucocorticoid-responsive genes located within 30 kbs from the GR-binding sites are transactivated by GR.

**Figure 3:**
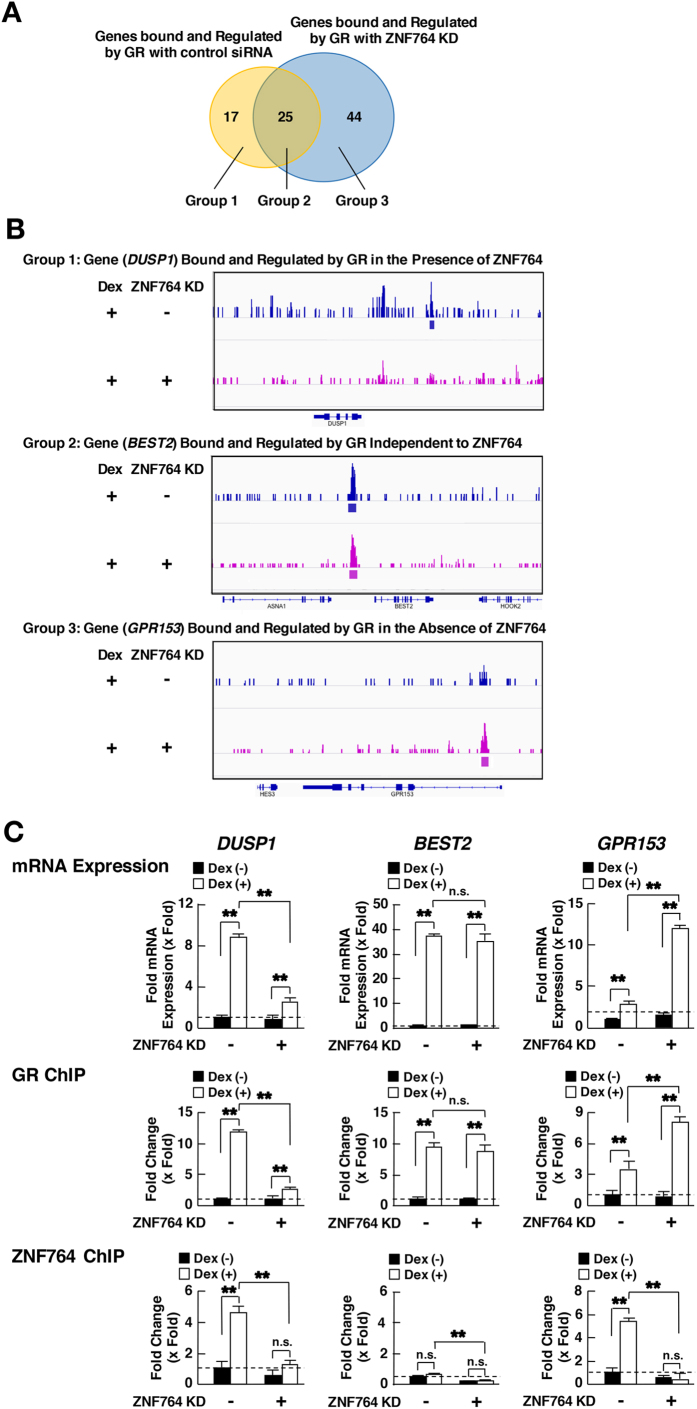
ZNF764 differentially regulates DNA-binding and transcriptional activity of GR. (**A**) Venn diagram for the genes differentially bound and regulated by GR in the presence or absence of ZNF764 knockdown (KD). Numbers of the GR-binding genes in the presence (right circle) or absence (left circle) of ZNF764 siRNA are shown. Group1: genes for which GR-binding sites are found only in the presence of ZNF764 (control KD), Group 2: genes for which GR-binding sites are found independently to ZNF764, Group 3: genes for which GR-binding sites are found only in the absence of ZNF764 (ZNF764 KD). (**B**) GR-binding profiles for the 3 representative genes. GR-binding profiles around the 3 representative genes bound and regulated by GR in the presence of (top: *DUSP1*), independent to (middle: *BEST2*) or in the absence of (bottom: *GPR153*) ZNF764 are shown. Statistically significant (p < 0.05) GR-binding sites are indicated with the colored boxes positioned under the corresponding peaks. (**C**) ZNF764 differentially regulates mRNA expression of the 3 representative genes and changes the attraction of GR and itself to GREs of these genes. HeLa cells were transfected with control or ZNF764 siRNA, and were cultured in the presence or absence of 10^−6^ M of dexamethasone. mRNA expression of *DUSP1, BEST, GPR153* and *RPLP0* was determined with the SYBR Green real-time PCR (top panels). Association of GR or ZNF764 to GREs of *DUSP1, BEST* or *GPR153* was determined using the ChIP assays performed using the anti-GR or -ZNF764 antibody and with the subsequent SYBR Green real-time PCR. Obtained ChIP signals were normalized for those with control IgG, and fold association was further calculated as the baseline with control siRNA in the absence of dexamethasone as “1” (middle and bottom panels). Bars represent mean ± S.E. values of fold *DUSP1* (left panel) *BEST2* (middle panel) or *GPR153* (right panel) mRNA expression normalized for *RPLP0* mRNA expression or fold association of GR or ZNF764 to GREs of these genes. **p < 0.01, n.s., not significant, compared to the conditions indicated. Broken lines indicate the level of fold expression as “1”.

We focused on the genes bound and regulated by GR. They were categorized into 3 groups with regard to their responsiveness to ZNF764 KD (Fig. 3A); Group 1: genes bound and regulated by GR strictly in the presence of ZNF764 (17 genes), Group 2: genes bound and regulated by GR independent to ZNF764 (25 genes) and Group 3: genes bound and regulated by GR strictly in the absence of ZNF764 (44 genes). The genes belonging to these 3 groups are listed in Supplemental Table 2. Figure 3B demonstrates the GR-binding profiles in their representative genes: Group 1: dual specificity phosphatase 1 (*DUSP1*), Group 2: bestrophin 2 (*BEST2*), and Group 3: G-protein-coupled receptor 153 (*GRP153*). GR bound *DUSP1* in the presence of ZNF764 but disappeared in its absence, while GR interacted with *GRP153* in the absence of ZNF764 but not in its presence. The GR bound *BEST2* regardless of the presence or absence of ZNF764. We evaluated mRNA expression of these genes in the presence or absence of ZNF764 using the SYBR Green real-time PCR (Fig. 3C, top panels). All 3 genes responded positively to dexamethasone, however the effect of this steroid varied greatly in the presence or absence of ZNF764, reflecting the binding profiles that we observed in ChIP-Seq. Dexamethasone strongly stimulated mRNA expression of *DUSP1* in the presence of ZNF764, but the effect was attenuated in its absence. *GRP153* mRNA demonstrated an opposite phenotype, responding strongly to dexamethasone in the absence of ZNF764, whereas its responsiveness to this steroid was significantly blunted in its presence. *BEST2* mRNA expression was highly induced by dexamethasone regardless of the presence or absence of ZNF764. Thus, ZNF764 regulates the responsiveness of the particular sets of glucocorticoid-responding genes by altering the binding of GR to the DNA sites of these genes. We also examined the association of GR or ZNF764 with the GREs of *DUSP1, BEST2* or *GRP153* using ChIP assays. We successfully identified the classic tandem GREs in the GR-binding sites of these 3 genes, and thus, performed the assays using the primer pairs that amplify the DNA fragments spanning these GREs (Supplemental Figure 2, and Supplemental Table 3). GR was associated with all three GREs in a dexamethasone-dependent fashion. The GR-binding profiles were consistent with the changes observed in the ChIP-Seq (Fig. 3C, middle panels); ZNF764 KD significantly attenuated and enhanced the GR-binding to the GREs of *DUSP1* and *GPR153* respectively, whereas it did not influence the binding to *BEST2* GREs. Interestingly, ZNF764 was attracted to the GREs of *DUSP1* or *GPR153* in a dexamethasone-dependent fashion in the absence of ZNF764 KD. In contrast, ZNF764 demonstrated residual binding to *BEST2* GREs, which was unresponsive to dexamethasone but still responsive to ZNF764 KD (Fig. 3C, bottom panels), suggesting that ZNF764 was not efficiently attracted to the GREs of this gene. Thus, the presence of ZNF764/GREs association underlies the successful regulation of ZNF764 on the GR-binding to GREs and its transcriptional activity on target genes regardless of the directions of its regulatory activities.

### ZNF764 and GR physically interact with each other through their KRAB domain and LBD

**Figure 4:**
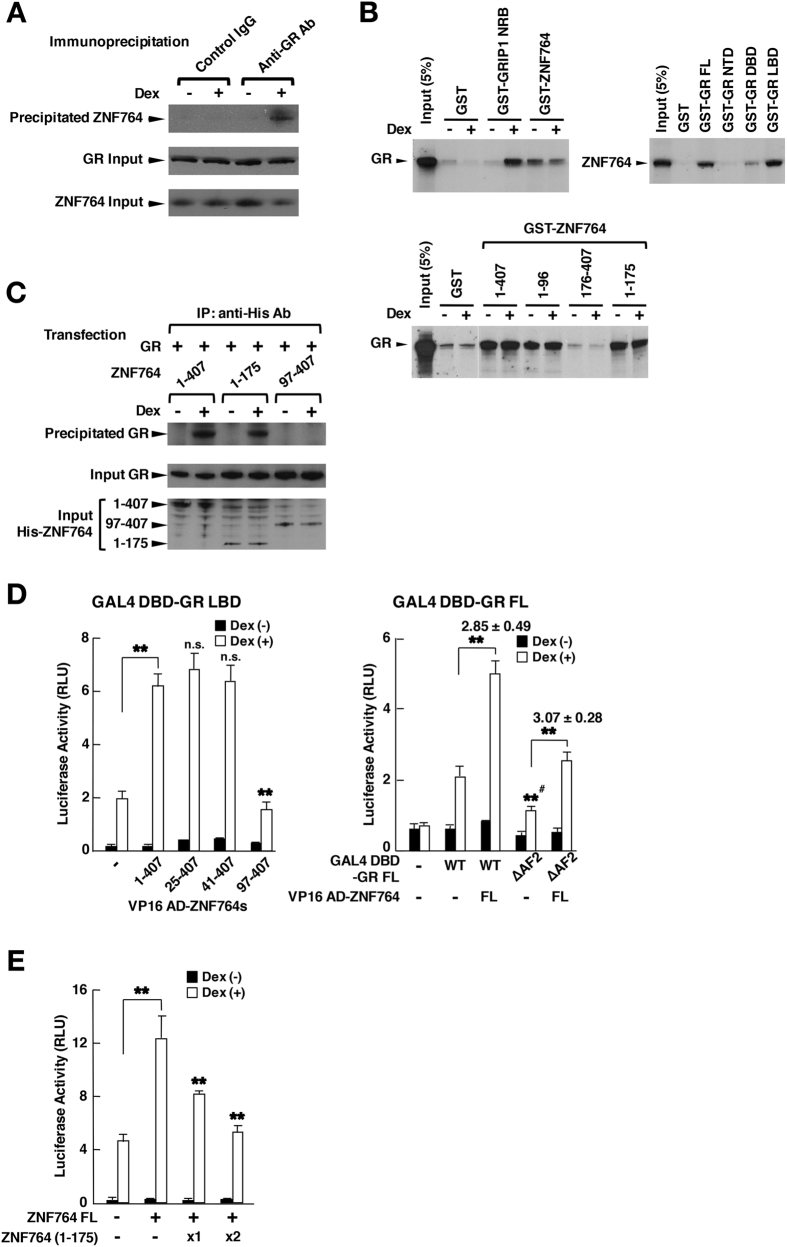
ZNF764 and GR physically interact through the former’s portion spanning amino acids 1-96 and the latter’s LBD. (**A**) ZNF764 is co-immunoprecipitated with GR in HeLa cells. HeLa cells were treated with 10^−6^ M of dexamethasone and immunoprecipitation was performed using anti-GR antibody or control IgG. Coprecipitated ZNF764 and ZNF764/GR in input samples were detected with Western blots. (**B**) ZNF764 and GR physically interact through the former’s portion spanning amino acids 1-96 and the latter’s LBD in GST pull-down assays. ^35^S-labelled GR or ZNF764 was incubated with indicated proteins fused with GST. NRB: nuclear receptor box (**C**) ZNF764 fragments containing amino acids 1-96 are co-precipitated with GR in HCT116 cells. HeLa cells were transfected with indicated His-ZNF764 fragment-expressing plasmid, and immunoprecipitation was carried out with anti-His antibody. Co-precipitated GR, and GR/ZNF764 fragments expressed in input samples were detected with Western blots. (**D**) ZNF764 and GR interact through the former’s amino acids 41-96 and the latter’s LBD outside the AF-2 surface in a mammalian two-hybrid assay. HCT116 cells were transfected with the plasmid expressing indicated VP16-AD-fused ZNF764 fragment and that expressing GAL4 DBD-GRα LBD (left panel) or -GRα full-length (FL) (right panel) together with GLA4 DBD-responsive p17mer-TK-Luc and pGL4.73[*hRluc*/SV40]. Bars represent mean ± S.E. values of the corrected firefly luciferase activity. In the right panel, numbers above the 2 bars indicate mean ± S.E. values of the fold association of the 2 molecules shown. **p < 0.01, n.s.: not significant, compared to the the two conditions indicated, or the condition in the presence of GAL4 DBD-GRα FL wild type (WT) and dexamethasone but in the absence of VP16 AD-ZNF764 (#). (**E**) GR-binding ZNF764 fragment (1-175) suppresses wild type ZNF764-induced enhancement of GR transcriptional activity in HCT116 cells. HCT116 cells were transfected with the plasmid expressing the indicated ZNF764-related molecules together with pRShGRα, pMMTV-Luc and pGL4.73[*hRluc*/SV40]. Bars represent mean ± S.E. values of the corrected firefly luciferase activity. **p < 0.01, compared to the the condition transfected with wild type (WT) ZNF764, but not its (1-175) fragment and in the presence of dexamethasone, or to the 2 conditions indicated.

Since attraction of ZNF764 to GREs appears to be important for its regulation on GR’s binding to GREs and its transcriptional activity, we examined the physical interaction between ZNF764 and GR in several assay systems (Fig. 4). In a co-immunoprecipitation assay using HeLa cells, ZNF764 was associated with GR in a dexamethasone-dependent fashion (Fig. 4A). In the glutathione-S transferase (GST) pull-down assays, GR bound GST-ZNF764 irrespective to the presence or absence of dexamethasone, whereas it interacted with the positive control GRIP1 NRB in this steroid-dependent manner (Fig. 4B, top, left gel). On the other hand, radio-labelled ZNF764 bound the full-length GR and its LBD fused with GST (Fig. 4B top, right gel). It is known that bacterially produced and GST-fused GR does not respond to glucocorticoids^39^,^40^. Using the several ZNF764 fragments fused with GST, GR bound the ZNF764 fragments containing amino acids 1-96, but not the fragment (176-407) that lacks this portion (Fig. 4B, bottom gel). These results indicate that ZNF764 and GR physically interact with each other through the former’s amino acids 1-96 and the latter’s LBD in a ligand-independent fashion in this assay system. In the co-immunoprecipitation assay employing His-tagged ZNF764 fragments, GR was co-precipitated with the full-length ZNF764 and its fragment (1-175) in a dexamethasone-dependent fashion, but not with the 97-407 fragment, indicating that GR physically interacts with ZNF764 at the latter’s portion spanning amino acids 1-96 in a dexamethasone-dependent fashion *in vivo* (Fig. 4C). Using the mammalian two-hybrid assay employing GAL4 DBD-fused GR LBD and VP16 activation domain (AD)-fused ZNF764, full-length ZNF764 and its fragments containing amino acids 41-407 interacted with GR LBD in a dexamethasone-dependent fashion, whereas the ZNF764 (97-407) fragment did not (Fig. 4D, left panel). Since the KRAB domain is located between amino acids 40-95, it is likely that ZNF764 binds GR through this motif. In another mammalian two-hybrid assay employing GAL4 DBD-fused wild type GR or its mutant defective in the AF2 surface (ΔAF2) and AV16 AD-fused full-length ZNF764, both these GR-related molecules bound ZNF764, suggesting that GR uses its LBD surface located outside the AF2, which is the transactivation domain created upon binding of GR LBD to ligand and supporting physical association between GR and the LxxLL motif of the p160-type nuclear receptor coactivators^40^ (Fig. 4D, right panel). This result is consistent with the fact that ZNF764 does not have a LxxLL motif. In a reporter assay employing the glucocorticoid-responsive mouse mammary tumor virus (MMTV) promoter-driven luciferase reporter gene, the ZNF764 (1-175) fragment having the binding site for GR suppressed the wild-type ZNF764-induced enhancement of GR transcriptional activity in a dose-dependent fashion (Fig. 4E), indicating that the ZNF764 (1-175) fragment acts as a dominant negative mutant for full-length ZNF764. This result further suggests that physical interaction between ZNF764 and GR is necessary for ZNF764 to enhance GR transcriptional activity on this promoter.

**Figure 5:**
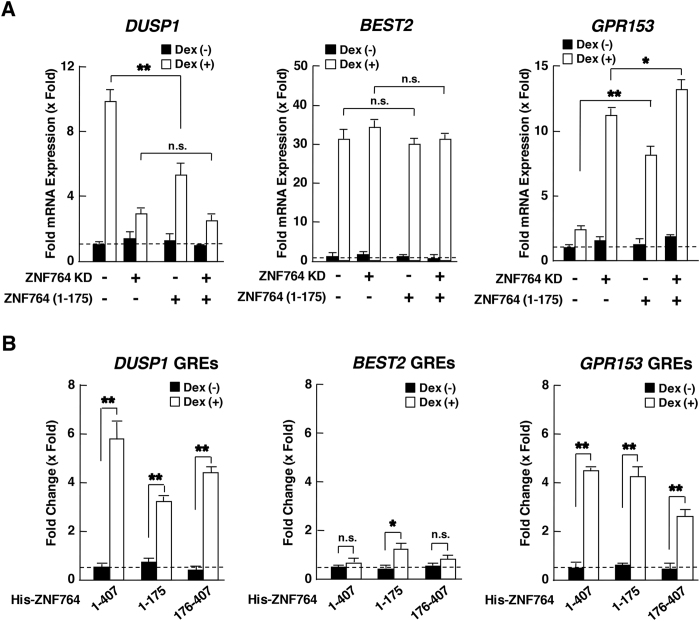
The KRAB domain and the zinc finger domain are both necessary for the regulation of GR transcriptional activity by ZNF764. (**A**) The transdominant negative fragment ZNF764 (1-175) competes with differential actions of endogenous ZNF764 on *DUSP1* and *GPR153*, but not on *BEST2*. HeLa cells were transfected with control or ZNF764 (1-175)-expressing plasmid in the presence or absence of control or ZNF764 siRNA, and were cultured in the presence or absence of 10^−6^ M of dexamethasone. mRNA expression of *DUSP1, BEST, GPR153* and *RPLP0* was determined with the SYBR Green real-time PCR using their specific primers. Bars represent mean ± S.E. values of fold *DUSP1* (left panel), *BEST2* (middle panel) or *GPR153* (right panel) mRNA expression normalized for *RPLP0* mRNA expression. Broken lines indicate the level of fold expression as “1”. *p < 0.05, **p < 0.01, n.s., not significant, compared to the 2 conditions indicated. (**B**) Both ZNF764 (1-175) and (176-407) fragments are attracted to *DUSP* and *GPR153* GREs, but not to *BEST2* GREs in HeLa cells. HeLa cells were transfected with the indicated His-tagged ZNF764 fragment-expressing plasmid, and were cultured in the presence or absence of 10^−6^ M of dexamethasone. Association of His-tagged ZNF764 fragments to GREs of *DUSP1, BEST* or *GPR153* was determined in the ChIP assays performed using the anti-His antibody and subsequent SYBR Green real-time PCR using specific primer pairs for these GREs. Obtained ChIP signals were normalized for those with control IgG, and fold association was further calculated as the condition transfected with ZNF764 (1-407) and in the absence of dexamethasone as “1”. Bars represent mean ± S.E. values of the fold association of the indicated ZNF764-related molecules to GREs of these genes. *p < 0.05, **p < 0.01, n.s., not significant, compared to the conditions indicated. Broken lines indicate the level of fold expression as “1”.

We therefore examined the effect of the ZNF764 (1-175) fragment on the 3 representative genes (*DUSP1, BEST2* and *GRP153*) differentially regulated by ZNF764 (Fig. 5). As expected, this dominant-negative ZNF764 fragment significantly reduced dexamethasone-induced *DUSP1* mRNA expression in the presence of endogenous ZNF764 but not in its absence (Fig. 5A). In contrast, this mutant strongly enhanced *GRP153* mRNA expression in the presence of dexamethasone and endogenous ZNF764, while its effect was significantly blunted in the absence of endogenous ZNF764. *BEST2* mRNA expression was not sensitive to this ZNF764 fragment at all. ZNF764 fragments spanning amino acids 1-175 and 176-407, which respectively contain the KRAB domain and zinc fingers were both attracted to *DUSP* and *GPR153* GREs similar to the full length ZNF764, whereas they were little or not attracted to *BEST2* GREs (Fig. 5B). Taken together, ZNF764 and GR physically interact with each other through the former’s KRAB domain and the latter’s LBD, and this physical interaction is necessary for ZNF764 to regulate GR transcriptional activity on both exogenous and endogenous glucocorticoid-responsive genes. Further, both KRAB domain and zinc fingers of ZNF764 appear to be required for its regulation of GR transcriptional activity, possibly by communicating with GR and/or cofactors through the former domain and by interacting with DNA through the latter. Association of ZNF764 to these GREs through its zinc fingers may underlie the dominant negative action of the ZNF764 (1-175) fragment harboring a KRAB domain.

### ZNF764 directs GR transcriptional activity toward distinct biologic pathways

**Figure 6:**
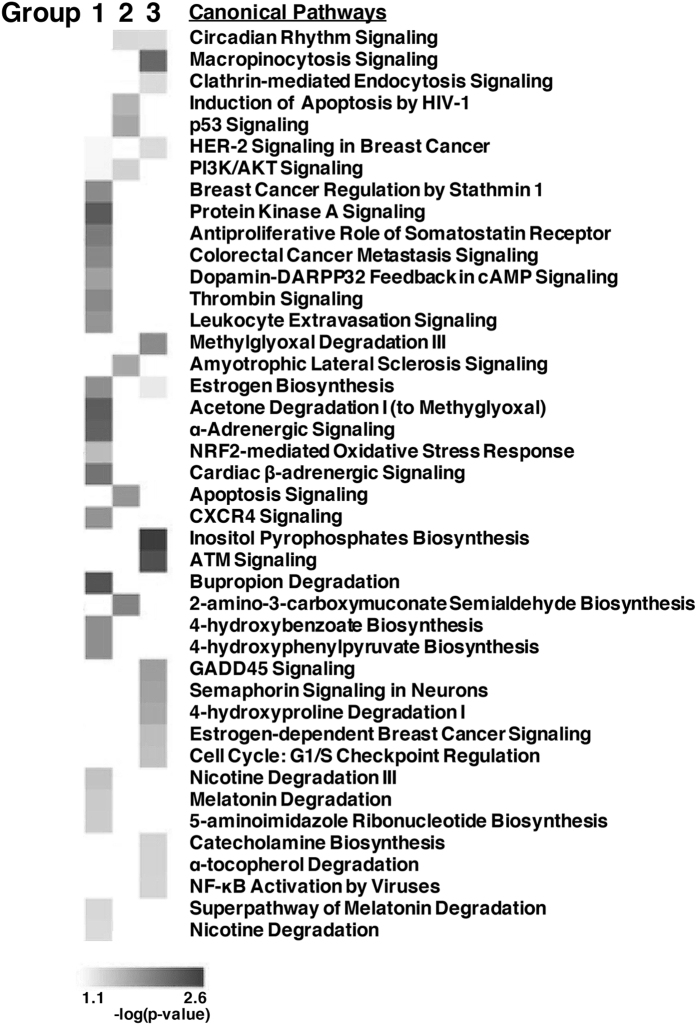
ZNF764 directs GR transcriptional activity toward distinct biologic pathways in HeLa cells. Results of the pathway analysis performed for the RNA-Seq data of the genes differentially bound and regulated by GR in the presence or absence of ZNF764 knockdown are demonstrated. Heat map for their expression is shown in the bottom. Group 1: genes bound and regulated by GR in the presence of ZNF764, Group 2: genes bound and regulated by GR independent to ZNF764, Group 3: genes bound and regulated by GR in the absence of ZNF764.

Since ZNF764 alters GR-binding and transcriptional activity of some glucocorticoid-responsive genes positively or negatively, we performed the pathway enrichment analysis for the 3 gene groups differentially responding to ZNF764 (Fig. 6). We found that they were enriched in different biological/signaling pathways; For example, Group 1 genes bound and regulated by GR in the presence of ZNF764 were highly associated with the pathways, such as those of the protein kinase A signaling, acetone degradation, α-adrenergic signaling and bupropion degradation. Group 2 genes unresponsive to ZNF764 were enriched in the pathways, including those associated with induction of apoptosis by HIV-1, p53 signaling, apoptosis signaling and 2-amino-3-carboxymuconate semialdehyde biosynthesis. Group 3 genes bound and regulated by GR in the absence of ZNF764 were enriched for the pathways, such as the macropinocytosis signaling, methylglyoxal degradation, inositol pyrophosphates biosynthesis and the ATM signaling. Thus, ZNF764 functions as a modulator of the GR transcriptional activity directing the actions of this receptor toward certain biologic pathways.

### ZNF764 does not change the affinity of GR to dexamethasone

It is known that New World monkeys living in Central or South America demonstrate pan-steroid hormone resistance^41^. For example, squirrel monkeys have almost 20-times higher levels of circulating cortisol due to significant reduction in the affinity of GR to this steroid^41^. These animals also demonstrate resistance to other steroid hormones, such as estrogen and progesterone^42^. We therefore expressed human or marmoset ZNF764 together with GR, and examined their impact on its affinity to radiolabelled dexamethasone in GR-deficient COS7 cells. Neither human nor marmoset ZNF764 changed the receptor affinity to this steroid in the whole cell dexamethasone binding assay (Supplemental Table 4). Further, these ZNF764s strongly and similarly enhanced GR transcriptional activity on the MMTV promoter in HCT116 cells (Supplemental Figure 3). Thus, ZNF764 does not influence the ligand-binding activity of GR and subsequent stimulation of the transcription of glucocorticoid-responsive genes.

## Discussion

In this manuscript, we examined details of the molecular interaction between GR and ZNF764, whose gene haploinsufficiency appears to be responsible for the multiple steroid hormone resistance observed in a patient with 16p11.2 microdeletion^18^. We found that ZNF764 and GR bound the genome region located distantly from TSSs of the nearby genes, and positioned very closely with each other. Indeed, ~37% of their binding sites overlapped. ZNF764 and GR physically interacted with each other through the former’s KRAB domain and the latter’s LBD in a glucocorticoid-dependent fashion. ZNF764 differentially modulated the association of GR to DNA and its transcriptional activity on the glucocorticoid-responsive genes functional in particular biologic pathways. Thus, ZNF764 functions as a modulator of glucocorticoid actions at target tissues, fine-tuning the transcriptional activity of GR.

Our pathway analysis provided some information to the biologic effects of ZNF764 on GR activity, but exact contribution of this molecule to the glucocorticoid/GR regulatory network is still largely unknown. In our previously reported case with 16p11.2 microdeletion, reduced sensitivity to glucocorticoids was observed in his HPA axis, particularly at the hypothalamic CRH/AVP neurons and pituitary corticotrophs^18^. Thus, glucocorticoid-responsive genes critical for this regulatory system appear to be potential targets of ZNF764. Although we did not examine the effects of ZNF764 on AR in this study, androgen-dependent male-type differentiation of fetal reproductive and urogenital tissues seems to be an important target of ZNF764, as our case demonstrated multiple birth defects in his external genitalia^18^. Examining the tissue/phase-specific ZNF764 expression and its effects on the GR/AR transcriptional activity may be helpful to elucidate the exact actions of ZNF764 on these receptors.

We found that ZNF764 binds DNA in close proximity to GR and modulates the association of GR to DNA and the transcriptional activity of GR on glucocorticoid-responsive genes. Physical interaction between ZNF764 1-97 (KRAB domain) and GR LBD is necessary for this regulation, and attraction of ZNF764 to GREs-bound GR appears to play roles both in the positive and negative regulation on the GR/GREs association and GR transcriptional activity in the 3 representative genes. Zinc fingers located in the C-terminal half of the ZNF764 may also contribute to this action of ZNF764 possibly through binding to DNA and/or other proteins. Thus, the ZNF764 that directly or indirectly binds DNA may communicate with the nearby GREs-bound GR, although it is still possible that the free ZNF764 binds GR through the direct protein-protein interaction to this receptor.

Exact mechanisms underlying the regulatory actions of ZNF764 on GR transcriptional activity are not known, but it is highly possible that the ZNF764 attracted to GR/GREs modulates the chromatin structure of its target glucocorticoid-responsive genes for both facilitation/suppression of their transcriptional activity. The KRAB domain is known to have chromatin modulatory activity by attracting KAP1, which further recruits the histone methyltransferase SETDB1, heterochromatin protein 1 (HP1) and the NuRD histone deacetylase complex 9^43^. This speculation is consistent with our previous finding that KAP1 (TIF1β) potentiates ZNF764-mediated enhancement of GR transcriptional activity on the MMTV promoter^18^. Further, the KRAB domain can function both as an enhancer and a repressor of transcription^44^. ZNF764 may also modulate the chromatin conformation through its zinc fingers. Indeed, ZNF366 with 11 zinc fingers without a KRAB domain represses the transcriptional activity of the estrogen receptor (ER) by communicating with a corepressor, the nuclear receptor-interacting protein 1, which attracts several chromatin modulatory proteins, such as histone deacetylases (HDACs) and the C-terminal tail-binding protein (CtBP)^45^. ZNF217 that contains seven C2H2-type zinc fingers without other characteristic protein motifs can attract chromatin/histone modulators, such as CtBPs, HDAC1/2 and histone demethylases LSD1 and Jarid1b/Plu-1^46^. In addition, ZNF764 might act as a modulator for the dynamic interaction of GR to GREs, possibly through the above-indicated chromatin modulatory activities. It is known that GR binds to/dissociates from GREs in a second order, and this characteristic GR/GREs association plays a key role in GR-mediated RNA polymerase II assembly, transcription initiation and mRNA elongation^47^,^48^. GR also has a function called “assisted reloading” with which pre-bound GR facilitates the access of the GR coming to GREs^49^. By modulating this dynamic nature of GR/GREs interaction, ZNF654 may be able to change the net GR accumulation to GREs, which is detected with ChIP assays.

As we encountered in the analysis on our ChIP-Seq data, identification of the binding motifs for KRAB-ZNFs is generally challenging, consistent with the fact that the binding motifs are not identified for the majority of these proteins^50^. This may be caused by the complicated character of KRAB-ZNF proteins in their interaction to DNA; Although these proteins directly bind their target motifs through their zinc fingers, they can also interact indirectly with DNA through other transcription factors and cofactors, which adds “noise” to the analysis on the ChIP-Seq data. Indeed, some KRAB-ZNFs are identified as components of the large coregulator complexes formed on the DNA-bound nuclear hormone receptors^51^. KRAB-ZNFs can bind RNA through their zinc fingers^26^,^28^,^29^, which also supports their indirect interaction to DNA. Further, the complexity in the way of sequence recognition by their zinc fingers may also contribute: multiple zinc fingers of the KRAB-ZNFs are selectively and/or differentially used in the recognition of different target sequences, resulting in providing a chance for one KRAB-ZNF to recognize multiple distinct motifs^23^.

We found that ZNF764 was associated with the motifs reported for other ZNF proteins (BCL6 and ZNF75-A) by employing highly confident binding sites. Thus, ZNF764 can share the sequence motifs with other C2H2-type zinc finger proteins, further indicating a possibility that ZNF764 modulates their activity by competing for the shared DNA-binding sites. Indeed, ZNF764 and BCL6 or ZNF75-A share 40–75% sequence homology in the amino acid sequences of their zinc fingers and some of them demonstrate 100% homology in their four key residues known to interact directly to DNA in triple-fingered ZNFs (data not shown). Since BCL6- and ZNF75-A-binding motifs were identified for the ZNF764-binding sites found respectively in the presence and absence of dexamethasone, it is possible that GR modulates the DNA-binding specificity of this protein. In addition, we found binding motifs for several C2H2-type ZNFs including BCL6 in the GR-binding sites found in the presence of dexamethasone, thus ZNF764 might use these motifs to come close to GR for some GR-binding sites.

Multiple steroid hormone resistance is attractive research area, but human cases harboring this pathologic phenotype are quite rare. Indeed, only one family demonstrating multiple steroid hormone resistance was reported prior to our case^16^,^17^, and the pathologic mechanism(s) for this family is (are) largely unknown. On the other hand, a physiologic form of multiple steroid hormone resistance is broadly recognized in New World monkeys in which reduction of receptor affinity to ligand plays a central role in its development, and several GR-interacting proteins are suggested to be involved in this condition^41^,^52^,^53^. We therefore examined the affinity of GR to dexamethasone in the presence or absence of ZNF764 overexpression. We found that both human and marmoset ZNF764 do not change GR affinity. Thus, ZNF764 is not a causative protein for the multiple steroid hormone resistance observed in New World monkeys. Future intensive research will warrant the area clarifying the molecular mechanisms underlying the multiple steroid hormone resistance.

## Materials and Methods

### Cell culture

Human cervical carcinoma HeLa and African green monkey kidney COS7 cells were maintained in Dulbecco’s modified Eagle Medium (DMEM) supplemented with 10% fetal bovine serum, 100 U/ml of penicillin and 100 μg/ml of streptomycin. Human colon cancer HCT116 cells were cultured in McCoy’s 5 A medium with the same supplements.

### Plasmids

pRShGRα and pGR107, which express human GRα respectively under the control of the Rous sarcoma virus promoter and the SP6 bacterial promoter, were gifts from Dr. R. M. Evans (Salk Institute, La Jolla, CA, USA). pM-GRα full-length (FL) and LBD, which express the corresponding portions of the wild type GRα fused with GAL4 DBD, were previously reported^4^,^39^. pM-GRα FL ΔAF2, which expresses the GAL4 DBD-fused GRα mutant defective in the AF2 surface^40^. pCDNA3.1 His/B-ZNF764 (1-407), (1-175) and (97-407), which express indicated portions of His-tagged human ZNF764, were created by inserting the corresponding portions of the human ZNF764 cDNA into pCDNA3.1 His/B (Invitrogen, Carlsbad, CA). pCDNA3.1 His/B-ZNF764 (Marmoset) was created by subcloning the marmoset ZNF764 cDNA obtained from B95 cells (American Type Cell Collection, Manassas, VA, USA) into pCDNA3.1 His/B. pGEX-4T3-GRIP1 NRB, -GRα FL, -GRα NTD, -GRα DBD and -GRα LBD, which express the corresponding portions of the mouse GRIP1 or the human GRα fused with GST were reported previously^11^,^39^. pGEX4T3-ZNF764 (1-407), (1-96), (1-175) and (176-407), which express the indicated portions of the human ZNF764 fused with GST, were created by inserting the corresponding portions of the human ZNF764 cDNA into pGEX-4T3 (GE Healthcare Bio-Sciences Corp., Piscataway, NJ, USA). pVP16-ZNF764 (1-407), (25-407), (41-407) and (97-407), which express the indicated portions of the human ZNF764 fused with the herpes simplex virus VP16 AD were created by subcloning the corresponding cDNA fragments into pVP16 (Clontech Laboratories, Inc., Mountain View, CA, USA). pMMTV-Luc and p17mer-TK-Luc, which express luciferase respectively under the control of the glucocorticoid-responsive MMTV promoter and the GAL4 response elements, were gifts from Drs. G. L. Hager (National Cancer Institute, Bethesda, MD, USA)^54^, and M. J. Tsai (Baylor College of Medicine, Houston, TX, USA), respectively. pGL4.73[*hRluc*/SV40], which expresses renilla luciferase under the control of the simian virus 40 promoter, was purchased from Promega Corp. (Madison, MI, USA).

### ChIP-Seq and ChIP assay

ChIP was performed by using the Chromatin Immunoprecipitation Assay Kit (Upstate, LCC., Lake Placid, NY, USA) in HeLa cells as previously reported^55^. Briefly, HeLa cells were grown on 10-cm dishes and were treated with 10^−6^ M of dexamethasone or vehicle ethanol for 2 hours. Some cells were transfected with either control or ZNF764 siRNA (Santa Cruz Biotechnology, Inc., Santa Cruz, CA, USA) using Lipofectamin 2000^®^ (Invitrogen, Carlsbad, CA, USA) prior to dexamethasone treatment. Cells were treated with 1% formaldehyde for 10 min to cross-link DNA and associated proteins, lysed with SDS lysis buffer (50 mM Tris-HCl [pH 8.1], 1% sodium dodecyl sulfate (SDS) and 10 mM ethylenediaminetetraacetic acid (EDTA)), sonicated to shear DNA using the Misonix Sonicator 3000 (QSonica, LLC., Newton, CT, USA), and the processed samples were resuspended in the ChIP dilution buffer (16.7 mM Tris-HCl [pH 8.1], 167 mM NaCl, 0.01% SDS, 1.1% Triton X-100, 1.2 mM EDTA, and protease inhibitors). They were then incubated with anti-GR or anti-ZNF764 antibody or control IgG overnight at 4 °C, and the antibody/protein/DNA complex was collected with the salmon sperm DNA/protein A agarose (Upstate, LLC.). The samples were washed with low salt immune complex wash buffer (20 mM Tris-HCl [pH 8.1], 150 mM NaCl, 0.1% SDS, 1% Triton X-100 and 2 mM EDTA), high salt immune complex wash buffer (20 mM Tris-HCl [pH 8.1], 500 mM NaCl, 0.1% SDS, 1% Triton X-100 and 2 mM EDTA), LiCl immune complex wash buffer (10 mM Tris-HCl [pH 8.1], 0.25 M LiCl, 1% IGEPAL-CA630, 1% deoxycholic acid and 1 mM EDTA), and 1X TE (10 mM Tris-HCl [pH 8.0] and 1 mM EDTA). The DNA/protein complex was finally eluted with the elution buffer (1% SDS and 0.1 M NaHCO_3_), and protein-DNA cross-link was reversed by incubating with 0.2 M NaCl at 65 °C for 4 hours. Liberated DNA was treated with 10 mM EDTA and 80 μg/ml of proteinase K (Sigma-Aldrich, St. Louis, MI, USA) at 45 °C for 1 hour and was precipitated with ethanol. Four batches of the DNA (5–10 ng) obtained in independent experiments were combined. Such samples were ligated with Illumina adaptors, subjected to 12 cycles of PCR to prepare final libraries, and were sequenced using the HiSeq 2000 System (Illumina, Inc., San Diego, CA, USA) at the DNA Sequencing and Genomics Core of the National Heart, Lung and Blood Institute (Bethesda, MD, USA).

ChIP assays for examining the association of GR or ZNF764 to the GREs of *BEST2, DUSP1* or *GRP153* were performed using anti-GR, -ZNF764 or -His antibody, or rabbit control IgG as indicate above, up to the step precipitating the chromatin DNA with ethanol. The DNA regions containing the GREs identified in the GR-binding sites of these genes (Supplemental Figure 2) were amplified from the prepared DNA samples using the primer pairs shown in Supplemental Table 3 in the SYBR Green real-time PCR using the 7500 Real-time PCR System (Applied Biosystems, Foster City, CA, USA), as previously described^40^. Amplification was performed in a two-step cycle: 95 °C for 10 min, then 45 cycles of the reaction consisting of denaturing at 95 °C for 15 sec and annealing/extension at 60 °C for 1 min. Obtained C*t* values were normalized for those of the corresponding inputs and those with control IgG, and their relative precipitation was demonstrated as fold precipitation above baseline.

### RNA sampling and sequencing (RNA-Seq)

HeLa cells were cultured as indicated for the ChIP-Seq experiment, and were treated with 10^−6^ M of dexamethasone or vehicle ethanol for 4 hours. Total RNA was then purified using the RNeasy Mini Kit (Qiagen, Hilden, Germany), and was subjected to the high-throughput RNA sequencing with duplicate.

### Analysis on ChIP-Seq or RNA-Seq Data

ChIP-Seq data were aligned to the reference hg19-GRCh37 assembly obtained from the UCSC Genome Browser (https://genome.ucsc.edu/cgi-bin/hgGateway?db=hg19) using the software Burrows-Wheeler Aligner (BWA)-MEM with default parameters (http://sourceforge.net/projects/bio-bwa/files/). Peak calling was performed with MACS2 (https://pypi.python.org/pypi/MACS2) by using its default settings (false discovery rate <5%), and fold-enrichment tracks (treatment vs. control) were generated with the bdgcmp tool of MACS2. Statistically significant binding peaks found in the ChIP-seq datasets were used for further analyses. We validated our ChIP-seq data by confirming the successful identification of GR or ZNF764 association to their top 10 binding sites found in the ChIP-seq in reconstituted ChIP assays. The closest genes to the identified binding peaks were determined with the peak2gene tool of Cistrome (http://cistrome.org/ap/), using the criteria that their transcription start sites (TSSs) are located within 30 kbs upstream or downstream of the respective binding peak. RNA-Seq data were aligned using TopHat (http://ccb.jhu.edu/software/tophat/index.shtml) with default parameters, and HTSeq (http://www-huber.embl.de/users/anders/HTSeq/doc/overview.html) was used to count reads aligned to each genomic feature. The output file was used to find differentially expressed genes in DESeq (http://bioconductor.org/packages/release/bioc/html/DESeq.html). Genes with an adjuested p value of < 0.05 were considered differentially expressed. Motif detection was performed with the SeqPos tool of Cistrome by searching in a region of 600 bps from the peak center. Visualization of peaks was carried out using the Integrative Genomics Viewer (IGV) genome browser (https://www.broadinstitute.org/igv/). Bed files for genome-binding profiles of the RNA polymerase II and p300 in HeLa cells were downloaded from ENCODE (http://www.genome.gov/encode/). BEDtools (https://github.com/arq5x/bedtools2/releases) were used to evaluate the distance between identified binding peaks and the closest TSSs or other target sequence motifs. The ChipSeq bam files for the different histones in HeLa cells were downloaded from ENCODE (H3K27ac: ENCFF000BBN, H3K4me3: ENCFF000BCO, H3K4me1: ENCFF000BBA, H3K27me3: ENCFF000BBS). Bedtools were used to extract the number of reads mapping to each base pair in each of either the GR- or ZNF764-binding peaks, and then normalized to the total number of reads in the corresponding bamfile. The plots were made using R (Quick-R, http://www.statmethods.net/advgraphs/layout.html). Pathway analysis was performed using the Ingenuity^®^ Pathway Analysis tool (Qiagen).

### Co-immunoprecipitation and Western blot

HeLa cells were treated with 10^−6^ M of dexamethasone or vehicle ethanol for 3 hours. Some cells were transfected with the indicated His-ZNF764-expressing plasmid and/or siRNA with Lipofectamine 2000^®^ prior to dexamethasone treatment. They were lysed in the buffer containing 50 mM Tris-HCl [pH 7.4], 150 mM NaCl, 0.1% SDS, 1% NP-40, 0.5% sodium deoxycholate and 1 Tab/50 ml Complete™ Tablet (Roche Diagnostics Corp., Indianapolis, IN, USA). Immunoprecipitation was carried out with anti-GR or -His antibody, or control IgG, and the protein complex was precipitated with protein A/G agarose (Santa Cruz Biotechnology, Inc.), as previously reported^56^. Samples were run on 4–12% Bis-Tris gels together with 3% of the input used for the co-immunoprecipitation reaction. Western blots were then performed for GR or ZNF764 using anti-GR, -ZNF764, -His or -β-actin antibody (Santa Cruz Biotechnology, Inc. or Sigma-Aldrich), and their protein bands were visualized using the horseradish-conjugated secondary antibody and the Amersham ECL Detection Reagents (GE Healthcare Bio-Science Corp.).

### GST pull-down assay

^35^S-labeled human GR and ZNF764 were generated with the *in vitro* transcription/translation reactions (Promega Corp.) using pR107 and pCDNA3.1His/B-ZNF764 as templates, respectively, and were tested for interaction with the GST-GRs or -ZNF764s immobilized on the glutathione-sepharose beads (GE Healthcare Bio-Science Corp.) in the presence or absence of 10^−5^ M of dexamethasone in the buffer containing 50 mM Tris-HCl [pH 8.0], 50 mM NaCl, 1 mM EDTA, 0.1% NP-40, 10% glycerol and 0.1 mg/ml BSA at 4 °C for 1.5 hour, as previously described^57^. Three μg of GST-fusion proteins were applied into each reaction. For the reactions employing GST-GRs, no dexamethasone was added, as these bacterially produced GR fusion proteins do not respond to glucocorticoids^39^,^40^. After vigorous washing with the buffer, proteins were eluted and separated on 8 or 4–12% Bis-Tris gels together with 5% of inputs used for the pull-down reactions. Gels were fixed, treated with Enlightening (NEN Life Science Products, Inc., Boston, MA), dried and exposed to film.

### Reporter assay

GR-deficient HCT116 cells cultured in 24-well plates were transfected with the indicated amounts (0–0.2 μg/well) of ZNF764- and GR-expressing plasmids together with 0.3 μg/well of pMMTV-Luc and 0.01 μg/well of pGL4.73[*hRluc*/SV40] using Lipofectamine 2000^®^ or the polyethylenimine (PEI) (Polysciences, Inc., Warrington, PA, USA)^40^. pCDNA3.1 His/B was used to keep the same amounts of transfected DNA. Six hours after the transfection, media were changed and cells were treated with 10^−6^ M of dexamethasone or vehicle ethanol for an additional 24 hours. Lysates were then analyzed for firefly and renilla luciferase activities using the Dual-Luciferase Assay Kit and the GloMax Luminometer (Promega Corp.), as previously reported^40^.

### Mammalian two-hybrid assay

Mammalian two-hybrid assay was performed as previously reported^58^. Briefly, HCT116 cells were transfected with 0.1 μg/ml of pM-GRα LBD, -GRα FL or -GRα FL ΔAF2, which respectively express GAL4 DBD-fused GRα LBD, full length GRα and its mutant defective in AF-2, and indicated amounts of the pVP16-ZNF764-related plasmids that express the corresponding portions of the ZNF764 fused with VP16 AD, together with 0.2 μg/ml of p17mer-TK-Luc and 0.01 μg/ml of pGL4.73[*hRluc*/SV40] by using Lipofectamine 2000^®^ in 24-well plates. pVP16 was used to keep the same amounts of plasmids. Twenty-four hours after transfection, 10^−6^ M of dexamethasone or vehicle ethanol was added to the medium. The cells were harvested after an additional 24 hours, and firefly and renilla luciferase assays were performed as described above.

### SYBR Green real-time PCR analysis

HeLa cells were transfected with control or pCDNA3.1 His/B-ZNF764 (1-175) using the the Nucleofector System (Lonza, Anaheim, CA, USA) with over 75% transfection efficiency. Twenty-four hours after the transfection, cells were treated with or without 10^−6^ M of dexamethasone, and were cultured for an additional 24 hours. Total RNA was then purified using the RNeasy Mini Kit (Qiagen). cDNA was synthesized using the TaqMan Reverse Transcription Reagents and oligo-dT as a primer (Applied Biosystems). PCR was performed in the 7500 Real-time PCR System (Applied Biosystems), as previously described^59^,^60^. Primer pairs for quantifying mRNA levels of *BEST1, DUSP1, GPR153* and *RPLP0* used in the SYBR Green real-time PCR were designed so that the sequence spanning between a forward and a reverse primer contains at least one intron. Their sequences are listed in Supplemental Table 5. C*t* values of the examined genes were normalized with those of the *RPLP0*, and fold changes were obtained by using the comparative C*t* method^61^.

### Statistical analysis

ChIP-Seq and RNA-Seq were performed as duplicate. All other experiments were performed as triplicate and were repeated at least 3 times, and their representative results were shown in Figures. Statistical analysis was performed by using the Student t-test with 2-tailed value in the GraphPad Prism 6 (GraphPad Software, San Diego, CA). Statistical significance was set at p < 0.05.

## Additional Information

**How to cite this article**: Fadda, A. *et al*. Genome-wide Regulatory Roles of the C2H2-type Zinc Finger Protein ZNF764 on the Glucocorticoid Receptor. *Sci. Rep.* **7**, 41598; doi: 10.1038/srep41598 (2017).

**Publisher's note:** Springer Nature remains neutral with regard to jurisdictional claims in published maps and institutional affiliations.

## Supplementary Material

Supplemental Materials

## Figures and Tables

**Table 1:** Numbers of the sites*/genes bound and regulated by GR in the presence or absence of ZNF764 knockdown (KD).

	w/o ZNF764 KD	w ZNF764 KD
Number of the GR-binding sites/genes regulated by GR	1,906/637	1,175/529
Number of the genes bound and regulated by GR	42	69
Number of the genes up-regulated by GR	37	67
Number of the genes down-regulated by GR	5	2

*Binding sites located less than 30 kb from TSSs of the closest genes are counted. w/o: without, w: with.
