# Supplementary material for: Genome-wide Regulatory Roles of the C2H2-type Zinc Finger Protein ZNF764 on the Glucocorticoid Receptor
Source: Sci Rep. 2017 Jan 31;7:41598. doi: 10.1038/srep41598 (PMC5282477; doi:10.1038/srep41598)
Supplement: Supplemental Materials [file srep41598-s1.pdf]

Supplementary Materials for

Genome-wide Regulatory Roles of the C2H2-type Zinc Finger Protein ZNF764 on the Glucocorticoid Receptor

Abeer Fadda[1], Najeeb Syed[2], Rafah Mackeh[1], Anna Papadopoulou[3], Shigeru Suzuki[3,4], Puthen V. Jithesh[2] and Tomoshige Kino[1,3]*

1: Division of Translational Medicine, Sidra Medical and Research Center, 2: Division of Biomedical Informatics, Sidra Medical and Research Center, Doha 26999, Qatar, 3: Program in Reproductive and Adult Endocrinology, *Eunice Kennedy Shriver* National Institute of Child Health and Human Development, National Institutes of Health, Bethesda, MD 20892, USA and 4: Department of Pediatrics, Asahikawa Medical University, Asahikawa 078-8510, Japan
*: Corresponding author

This file includes:

METHOD USED FOR SUPPLEMENTAL MATERIALS

REFERENCES FOR SUPPLEMENTAL MATERIALS

Supplemental Table S1. Quality of the representative ChIP data used in this study.

Supplemental Table S2. Influence of ZNF764 on the genes bound and regulated by GR.

Supplemental Table S3. Primers for examining the association of GR or ZNF764 to GREs of the 3 representative genes in SYBR Green real-time PCR.

Supplemental Table S4. The effect of human or marmoset ZNF764 on the affinity of GR to dexamethasone in COS7 cells.

Supplemental Table S5. Primers for examining mRNA expression of the 3 representative genes in SYBR Green real-time PCR.

Supplemental Figure S1. ZNF764 siRNA significantly reduces ZNF764 protein expression in HeLa cells.

Supplemental Figure S2. Sequences of the GR-binding sites associated with *DUSP1*, *BEST2* or *GPR153*.

Supplemental Figure S3. Human and marmoset ZNF764 similarly and strongly enhances GR transcriptional activity.

METHOD USED FOR SUPPLEMENTAL MATERIALS

Whole cell dexamethasone binding assay

GR-deficient COS7 cells were seeded into 12-well plates, and were transfected with 0.5 μg/well of the indicated ZNF764-expressing plasmid or control pCDNA3.1 His/B together with 0.5 μg/well of pRShGRα. One day after the transfection, medium was replaced with DMEM without FBS supplementation, and whole cell dexamethasone binding assays were performed as previously described (1). Briefly, cells were incubated with grading concentrations of [1,2,4,6,7-^{3}H] dexamethasone (GE Healthcare Bioscience Corp., Piscataway, NJ, USA) in the presence or absence of 500-fold higher concentrations of cold dexamethasone for 90 min. After vigorous washing with PBS, cells were harvested by treating with trypsin, washed once with PBS, and radioactivity associated with harvested cells was counted with the LS6000IC Scintillation Counter (Beckman Coulter, Inc., Fullerton, CA, USA). Scatchard analysis was performed with the GraphPad PRISM Version 6 (GraphPad Software, San Diego, CA) for evaluating affinity (Kd) to dexamethasone.

Western Blot and SYBR Green real-time PCR analysis

Please see manuscript text.

REFERENCES FOR SUPPLEMENTAL MATERIALS

1. Nader, N., Bachrach, B. E., Hurt, D. E., Gajula, S., Pittman, A., Lescher, R., and Kino, T. (2010) A novel point mutation in helix 10 of the human glucocorticoid receptor causes generalized glucocorticoid resistance by disrupting the structure of the ligand-binding domain. *J Clin Endocrinol Metab* **95**, 2281-2285

Supplemental Table S1. Quality of the representative ChIP data used in this study.

File Name	Total Number of Aligned Reads	Number of Reads in Peaks	FRiP (%)
ChIP using anti-ZNF764 antibody on the cells without Dex	26,630,643	711,832	3.0
ChIP using anti-ZNF764 antibody on the cells with Dex	20,198,148	267,892	1.0
ChIP using anti-GR antibody on the cells with control siRNA and Dex	11,300,756	395,549	3.5
ChIP using anti-GR antibody on the cells with ZNF764 siRNA and Dex	13,412,334	233,998	1.7

FRiP: Fraction of Reads in Peaks. Dex: Dexamethasone

Supplemental Table S2: Influence of ZNF764 on the genes bound and regulated by GR.

All genes	Genes found in the presence of ZNF764	Genes independent to ZNF764	Genes found in the absence of ZNF764
DUSP1	DUSP1	ADAMTSL4	CDKN1C
EDN2	EDN2	ANKRD33B	FKBP5
PPARGC1B	PPARGC1B	ANPEP	GPR153
SLC19A2	SLC19A2	B4GALT1	MT2A
BCAR3	BCAR3	BCL2L1	MT1E
KCNIP3	KCNIP3	BEST2	PNMT
ACTC1	ACTC1	BIRC3	NFKBIA
NR2F1	NR2F1	C10orf10	KCNJ11
SLC45A4	SLC45A4	DDIT4	NEBL
CXCR4	CXCR4	ETNK2	ADAMTS7
F2R	F2R	FAM129B	TENC1
DOCK11	DOCK11	FAM83A	ABLIM3
TLR6	TLR6	HIF3A	JUNB
STEAP4	STEAP4	HSPB7	PSCA
ARL4D	ARL4D	ITGB4	STAC2
ADARB1	ADARB1	KRT5	CDKN1A
TBX15	TBX15	KRT6A	ITGA5
ADAMTSL4		MCL1	PYGB
ANKRD33B		PER1	KLF6
ANPEP		RASSF4	A4GALT
B4GALT1		SALL4	IP6K3
BCL2L1		SCNN1A	IL6R
BEST2		SLC39A14	RHOB
BIRC3		TFCP2L1	PACSIN2
C10orf10		ZMYND8	PIM3
DDIT4			TSPYL2
ETNK2			PKP2
FAM129B			MFGE8
FAM83A			FIBCD1
HIF3A			TOP1
HSPB7			MT1M
ITGB4			ZBTB7B
KRT5			GGT5
KRT6A			TCAP
MCL1			BEST1
PER1			JRK
RASSF4			MYOCD
SALL4			ARF6
SCNN1A			WDR72
SLC39A14			MT1A
TFCP2L1			CDA
ZMYND8			DECR1
CDKN1C			CHST7
FKBP5			GIPC1
GPR153			
MT2A			
MT1E			
PNMT			
NFKBIA			
KCNJ11			

Continuing

All genes	Genes found in the presence of ZNF764	Genes independent to ZNF764	Genes found in the absence of ZNF764
NEBL			
ADAMTS7			
TENC1			
ABLIM3			
JUNB			
PSCA			
STAC2			
CDKN1A			
ITGA5			
PYGB			
KLF6			
A4GALT			
IP6K3			
IL6R			
RHOB			
PACSIN2			
PIM3			
TSPYL2			
PKP2			
MFGE8			
FIBCD1			
TOP1			
MT1M			
ZBTB7B			
GGT5			
TCAP			
BEST1			
JRK			
MYOCD			
ARF6			
WDR72			
MT1A			
CDA			
DECR1			
CHST7			
GIPC1			

Supplemental Table S3. Primers for examining the association of GR or ZNF764 to GREs of the 3 representative genes in SYBR Green real-time PCR.

Gene Name		Primer Sequence (5' to 3' orientation)
**Human**		
BEST2	Forward	CCTCCCACACCAATCTG
	Reverse	GCATTTGGCATGGATTCTG
DUSP1	Forward	GAGCCCAGAGAGGTTAGG
	Reverse	GTTTCTATTTGTGACTTAAGG
GPR153	Forward	GACAGAGGACTGGTTGTG
	Reverse	CGCTCCATTCTATGAAG

BEST2: bestrophin 2, *DUSP1*: dual specificity phosphatase 1, *GPR153*: G protein-coupled receptor 153

Supplemental Table S4. The effect of human or marmoset ZNF764 on the affinity of GR to dexamethasone in COS7 cells.

		Control	Human ZNF764	Marmoset ZNF764
**Kd (nM)**				
	Mean	2.17	1.65	2.06
	S.E.	0.519	0.183	0.060
	P values*		0.396	0.838
**Bmax (x10^{-10} nmol/cell)**				
	Mean	9.03	5.50	1.20
	S.E.	1.452	0.402	0.598
	P values*		0.079	0.132

*: P values were obtained by comparing to control.

Supplemental Table S5. Primers for examining mRNA expression of the 3 representative genes in SYBR Green real-time PCR.

Gene Name		Primer Sequence (5' to 3' orientation)
**Human**		
BEST2	Forward	GGAGCATCTACAAACTC
	Reverse	CATACTGGTCACAATAAATCAC
DUSP1	Forward	CAAGTCTTCTTCCTCAAAGG
	Reverse	GAACTGCACCCAGATTCC
GPR153	Forward	CTCTCCTACCACCGCATGTG
	Reverse	CACCATCCAGATACCCATGAC
RPLP0	Forward	GAGGACCTCACTGAGATTCG
	Reverse	CTGGAAGAAGGAGGTCTTCTC

BEST2: bestrophin 2, *DUSP1*: dual specificity phosphatase 1, *GPR153*: G protein-coupled receptor 153, *RPLP0*: acidic ribosomal phosphoprotein P0

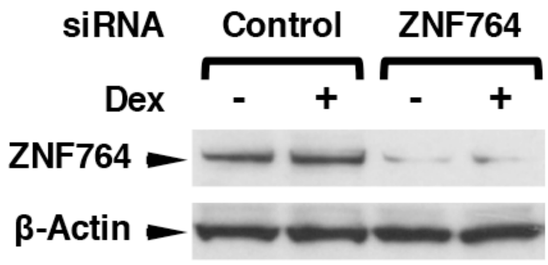

Supplemental Figure S1. ZNF764 siRNA significantly reduces ZNF764 protein expression in HeLa cells. HeLa cells were transfected with control or ZNF764 siRNA and treated with 10^{-6} M of dexamethasone. Cells were lysed and Western blots were performed using anti-ZNF764 or -β-actin antibody.

DUSP1 GR-binding Site (Chromosome 5: 172,202,751-172,202,844)

AAGAGCCCAGAGAGGTTAGGCAATCTGTCCAAA<u>**GTCACACAGTGTTCAG**</u>AACATT
CTAACCGTAGTCAGCATTGTGTGAGCTGTGTGTCCAAACCCTTAAGTCACAAATAG
AAACC

BEST2 GR-binding Site (Chromosome 19: 12,861,106-12,861,472)

CTGGACAGCGAAGGCATGGGTCACAGGGTGACCAGCCCAGGCGGGTTCCTAATT
ACTCCGGGCAGGGTGAACATCTGCCAGTCTGGGAACACGGCGTCCCAGCCTGCT
GGGCTTGGAACCTCGGGCGGGGCTATGTAGAATGCACGAGGGGCTCCTGAGCAC
TCTAGGATGCCCATCCCAGGGTTTGACCCCTGAACTTCTGCCCCTCCCACACCAAT
CTGTGTGTTAATGTTCTTCAGGCTGGGCAATGGAGGGGGAGT<u>**GGGTACTGTTTGT**</u>
<u>**AC**</u>CAGGCTGGGAGTGTCCCCTTTAGAGGTGTGTCTGTGAAGAATGAACATCTGCC
CAGAATCCATGCCAAATGCAGGACAAGGTTTTGTGTCCC

GPR153 GR-binding Site (Chromosome 1: 6,319,798-6,319,985)

TTCCAAGTGCAGAGTGACAGAGGACTGGTTGTGTG<u>**GGAACAAAGTGTCCCTC**</u>TGG
CTCTGGGTCAGTGACCACAGATCCACGGATTGCACCCTTTCTGTTCATTCTTCATA
GAATGGAGCGGCCACTAGGGCCAGACCCCAGGCTGGGCTCCCCACAGTGCCGAC
GGGACCCAGTCTCCTGCTGGGAAGTGTCTCCTGCAGGGCACTT

Supplemental Figure S2. Sequences of the GR-binding site associated with *DUSP1*, *BEST2* or *GPR153* found in the ChIP-Seq examination.
Classic, tandem GREs found in the GR-binding sites associated with *DUSP1*, *BEST2* or *GPR153* are shown in bold and with underline. The numbers after "Chromosome" indicate locations of the shown sequences in the hg19-GRCh37 assembly.

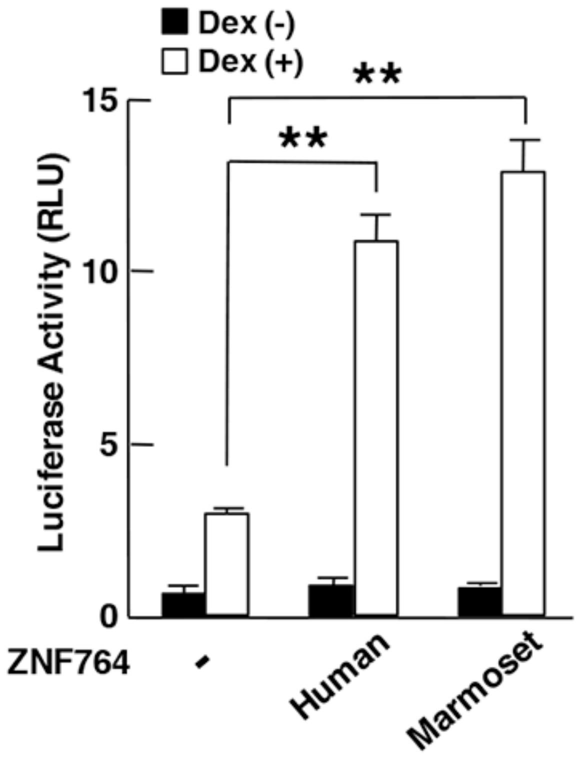

Supplemental Figure S3. Human and marmoset ZNF764 similarly and strongly enhances GR transcriptional activity.

HCT116 cells were transfected with the plasmid expressing human or marmoset ZNF764 together with pRShGRα, pMMTV-Luc and pGL4.73[*hRluc*/SV40], cultured in the presence or absence of 10^{-6} M of dexamethasone, and firefly and renilla luciferase assays were performed. pCDNA3.1 His/B was used as a control for ZNF764-expressing plasmids. Bars represent mean ± S.E. values of firefly luciferase activity normalized for renilla luciferase activity. **: p<0.01, compared to the the 2 condition indicated.
